# Hydrodynamic performance of floating photobioreactors driven by wave energy

**DOI:** 10.1186/s13068-019-1396-9

**Published:** 2019-03-16

**Authors:** Chenba Zhu, Zhanyou Chi, Chunwei Bi, Yunpeng Zhao, Haibo Cai

**Affiliations:** 1 School of Life Science and Biotechnology, Dalian University of Technology, Dalian, 116024 China; 2 State Key Laboratory of Coastal and Offshore Engineering, Dalian University of Technology, Dalian, 116024 China; 3 State Key Laboratory of Biotechnology, East China University of Science and Technology, Shanghai, 200237 China

**Keywords:** Floating PBR, Scaling-up, Wave, Hydrodynamic movement

## Abstract

**Background:**

Unlike conventional cultivation systems, liquid mixing in floating photobioreactors (PBRs) is solely induced by their hydrodynamic movement in response to waves, and this movement is affected by the wave conditions (wave height and wave period), the PBR configuration and the culture depth. However, to the best of our knowledge, a practical study of the hydrodynamic movements of PBRs has not been previously conducted.

**Results:**

This study aims to investigate the hydrodynamic performance of floating PBRs in response to wave conditions. First, the effects of the experimental wave height (2–10 cm) and wave period (0.8–1.8 s) on movement was investigated using two 1.0 m^2^ PBR models: a square PBR (1.0 m/1.0 m; length/width) and a rectangular PBR (1.7 m/0.6 m). The results indicated that wave movement became not only more intense with increasing wave height, but also less intense when the wave period decreased. However, the square PBR experienced more intense movement than the rectangular PBR, but also little mooring force. The effects of culture depth (0.5, 1.0 and 2.0 cm) were investigated and the results showed that the culture depth significantly affected the hydrodynamic movements of the PBRs; however, the mooring forces were unaffected. Finally, the movement and mooring-line forces of PBRs equipped with different mooring systems were investigated. The use of two different mooring systems had little effect on PBR movement; however, a mooring system with floaters was able to significantly reduce the mooring line forces compared to a system without floaters. During this study, the greatest force (10.5 N) was found for the rectangular PBR using a mooring system without floaters, whereas the lowest force (0.67 N) was observed for a rectangular PBR using a mooring system with floaters.

**Conclusions:**

These studies have provided basic data describing the fluid dynamics of floating PBRs; as well as their structural design and scale up. These results also provide guidance for the selection of ocean fields with suitable wave conditions; as well as a proper mooring methods to ensure safe operation.

## Background

Microalgae have been recognized as one of the most ancient living microorganisms. Microalgae have a rapid growth rate which is 100 times faster than terrestrial plants [1]; as well as a higher lipid content which makes them ideal for biodiesel production. Microalgae also have higher efficiency than plants for capturing CO_2_ from the atmosphere. Microalgae are also more efficient at capturing solar energy than are terrestrial plants [2] (i.e., rates of 10–50 times greater) [3]. In addition, the production of their biomass does not compete with other uses of arable land and food supplies [4, 5]. Moreover, the photosynthetic efficiency of microalgae is higher than that of land plants; the theoretical maximum of their photosynthetic efficiency is approximately 10% [1]. Due to these advantages, microalgal biomass has been established as one of the most promising sources of biofuel feedstock today [6, 7]. Presently, open ponds, which require little capital and have low operating costs, are widely used in commercial biomass production; however, they are also problematic due to low biomass productivities and a high risk of culture contamination [8]. Compared to open ponds, closed photobioreactors (PBR) have higher biomass productivity and also considerably higher production costs due to the high energy required for their operation and maintenance, as well as high capital costs [9, 10]. Thus, it is of interest to develop PBRs with lower capital and operation costs for use in the future.

Currently, considerable effort has been employed to reduce PBR costs, including the development of new types of PBRs, such as the Taylor vortex algal PBRs [11], membrane PBRs [12, 13], and the improvement of biomass productivity by optimizing PBR construction using computational fluid dynamics (CFD) simulations [14, 15]. Among the designs that have been created, floating PBR systems represent an emerging technology that has significant advantages over traditional PBR systems, as it utilizes ocean waves to provide free energy for mixing [16], the surrounding water to control the temperature [17], and the open ocean to provide low cost space, nutrients and water for algae growth [18]. In our previous study, a floating cultivation system known as the Bicarbonate-based Carbon Capture and Algal Production System on Ocean (BICCAPSO) was developed [16, 19, 20], in which inorganic carbon was supplied via bicarbonate to develop a simple PBR that did not require aeration and agitation with the aim of reducing the costs of PBR manufacture and installation. During outdoor culturing in the ocean, the biomass productivity of BICCAPSO can yield up to 18.9 g m^−2^ day^−1^, which indicates that this system can effectively produce microalgal biomass.

Mixing is obligatory for microalgae cultivation, as it not only enhances mass transfer, but also improves the frequency in the shifts between dark and light of algal cells, which leads to high photosynthetic efficiency and biomass productivity [21]. Commonly, biomass productivity increases as the mixing rate is increased [22], as long as the increased mixing does not damage algae cells. However, an increased mixing rate also significantly increases operation costs [23–25], especially for conventional closed PBRs. In contrast to a conventional closed PBR, mixing in a floating PBR can utilize energy provided by free waves. Nevertheless, most floating systems that have been developed to date have utilized the same mixing devices as land-based PBRs, including gas bubbling systems [26], paddlewheels [27] and circulation pumps [28]. Apart from the devices’ intensive energy requirements, these mixing devices also limit the potential for scale-up of PBR-based cultivation [29]. Moreover, mixing devices would likely cause serious operation problems in the unpredictable environment of the ocean [16]. Despite the existence of an extensive body of literature regarding floating PBRs [16, 19, 28, 30, 31], studies of mixing in floating PBRs and the effects of mixing on PBR performance have been limited. There have been some reports of floating PBRs with internal partitions [26], in which the mass transfer of O_2_ (k_L_a_O2_) was characterized and reported; however, the mixing in these PBRs was performed using air bubbling. Compared with other floating systems, BICCAPSO does not have any auxiliary mixing device, and mixing or liquid sloshing is solely the result of its movements in response to waves. Hence, to study mixing in BICCAPSO, it is necessary to study the movement of floating PBRs in response to waves as a first step, as this movement is induced and affected by wave conditions.

The floating PBR can be treated as a typical floating structure that is similar to other offshore structures used in ocean engineering, such as the floating breakwater [32], floating oil boom [33], and LNG-FPSO ship [34]. Currently, the physical model is widely used to investigate the hydrodynamic movement of offshore equipment [34, 35]. According to the previous studies, wave conditions and structure are the two basic factors that affect the movement responses of floating objects, which should also be true for floating PBRs. Culture depth is another significant factor that affects not only movement responses, but also the average light intensity, which in turn affects the photosynthetic efficiency of microalgae. The aim of this study was to investigate the movement of floating horizontal PBRs in response to waves using a physical model of a 1.0 m^2^ PBR at a geometrical similarity scale of 1:10. To study the movements in response to waves, three scenarios were considered: (i) the effects of wave parameters, including wave height (0.2–1.0 m) and wave period (2.53–5.69), (ii) the effects of the aspect ratio of the PBR, and (iii) the culture depth. Additionally, the effects of the mooring method on hydrodynamic performance and the mooring line force were measured to develop a proper mooring system that can be adjusted for different wave conditions.

## Methods

The objective of the present study was mainly to focus on the motion and load of the floating PBR in the presence of waves. The study was conducted with a downscaled reactor model, a method that has been widely used for the study of other ocean floating objects, such as floating breakwaters [32], floating oil booms [33] and liquefied natural gas-floating production, storage and offloading system (LNG-FPSO) ships [34]. As both gravity and inertial forces are key factors that determine the wave load, the floating PBR used in the physical model experiment should satisfy the Froude similarity criterion to guarantee that the prototype and the model both have the same Froude number. In addition, the motion and load of floating PBRs show periodic changes in waves. Thus, the Strouhal number of the prototype and the model should be consistent to satisfy the Strouhal similarity criterion [36]. Based on the similarity criteria, the transformation relationships between the various physical parameters of the model and the prototype are shown in Table 1; a geometrical similarity scale of 1:10 was used in this study.

**Table 1:** Transformation relationships between various physical parameters of the model and the prototype

Physical parameter	Transformation coefficient (Prototype: model)
Acceleration	1
Area	*λ* ^2^
Angle	1
Depth	*λ*
Force	*λ* ^3^
Mass	*λ* ^3^
Velocity	*λ* ^1/2^
Volume	*λ* ^3^
Wave height	*λ*
Wave length	*λ*
Wave period	*λ* ^1/2^

**Fig. 1:**
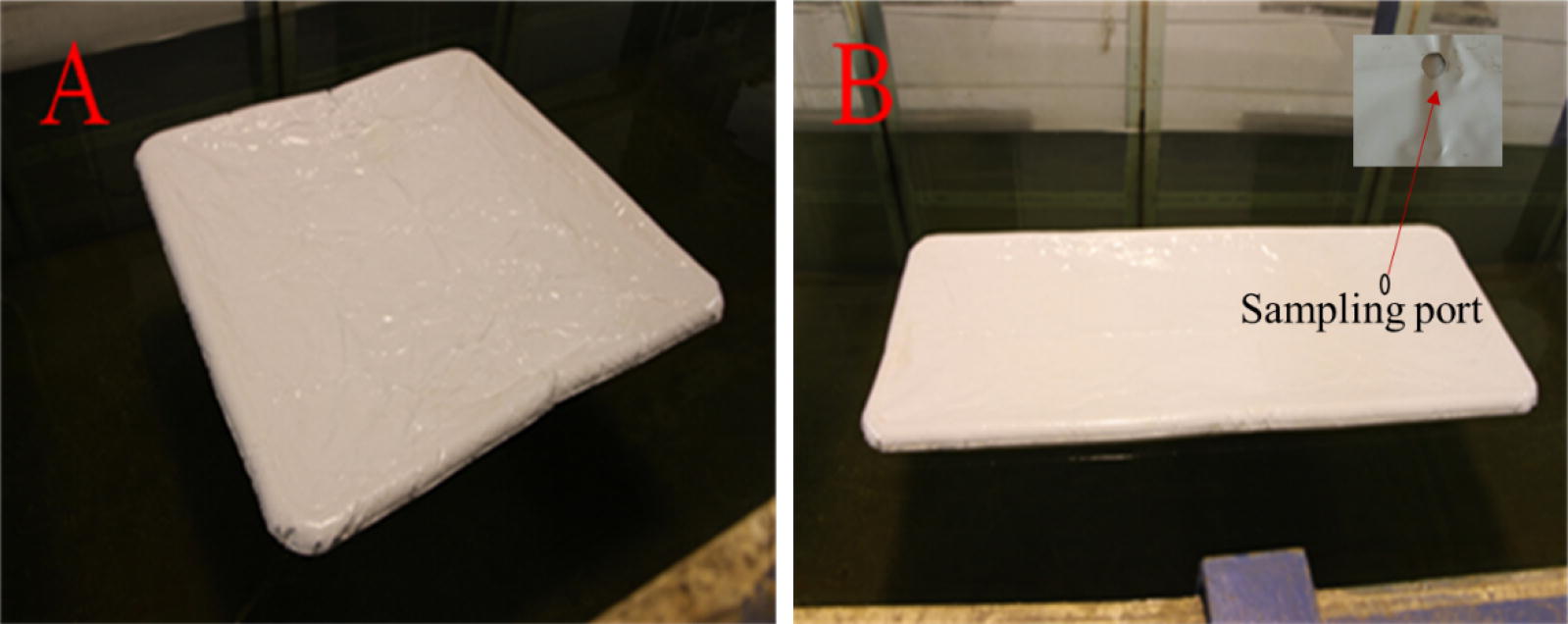
The 1.0 m^2^ floating PBR models: **A** square and **B** rectangle PBR

To investigate the hydrodynamic performance of the floating PBR, a series of laboratory experiments were conducted. All laboratory experiments were performed in a wave-current flume with a size of 69:2:1.8 m (length/width/height; Fig. 1) at the State Key Laboratory of Coastal and Offshore Engineering, Dalian University of Technology, Dalian, China.

### PBR models

**Fig. 2:**
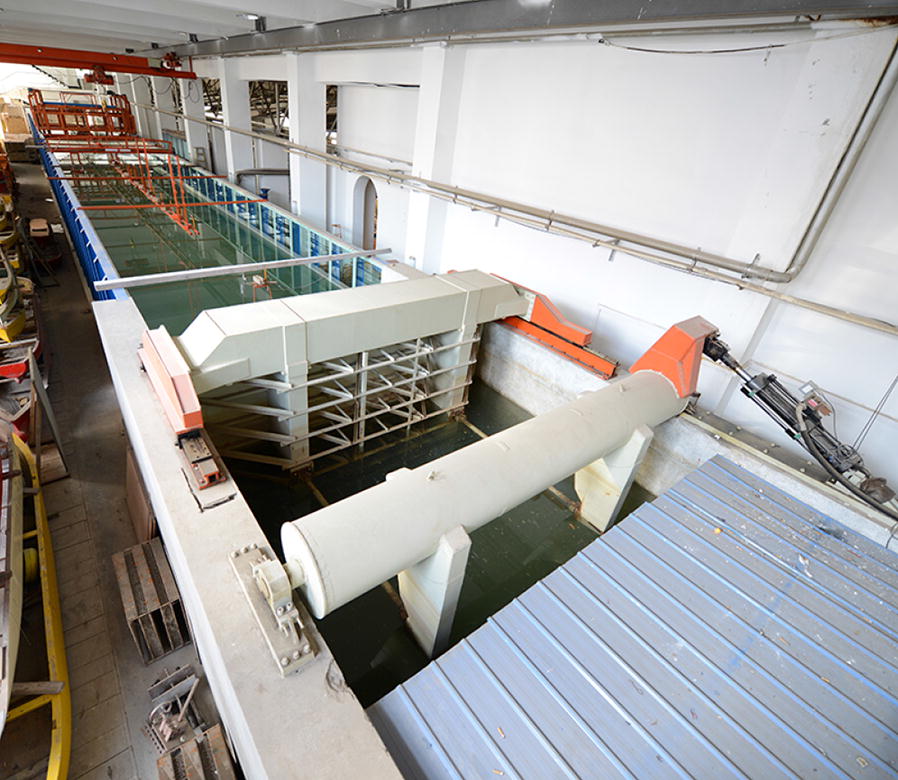
Diagram showing the water tank that used for testing the hydrodynamics of floating PBRs

Two inflatable PBRs (square and rectangular in shape) were used for the experiments (Fig. 2). The dimensions of the square PBR were 112 cm × 112 cm × 6.0 cm (length/width/height), whereas the rectangular PBR had dimensions of 182 cm × 72 cm × 6.0 cm (length/width/height). Both had an effective area of 1.0 m^2^, whereas the corresponding floating PBR prototype had an area of 100 m^2^. A polyvinyl chloride (PVC) membrane was used to make the inflatable PBR. A non-transparent PVC inflatable tube was used for the frame of the PBR, and the size of the inflatable pipe was 6.0 cm. The PBRs were covered with PVC and sealed, with only a sampling port left open on the top of the membrane surface that could be used for pumping water into the PBR or drawing water from the PBR. There are four beckets on the inflated tube that were used for anchoring the PBR in water. Air could be blown into an inflatable tube through a valve to maintain a firm structure, and it was assumed that this structure did not generate permanent deformation during the testing process.

### Experimental setup

The water depth (*h*) of the wave-current flume used during the experiments was 1.0 m and all tested waves and currents used in the present study could be generated using this flume. The flume is equipped with a servo-motor-driven, piston-type wave maker capable of producing regular and irregular waves. At the other end of the flume, wave absorbers are installed to mitigate wave reflection. Both sides of the flume within the working section are smooth glass to reduce viscous dissipation due to boundaries.

**Fig. 3:**
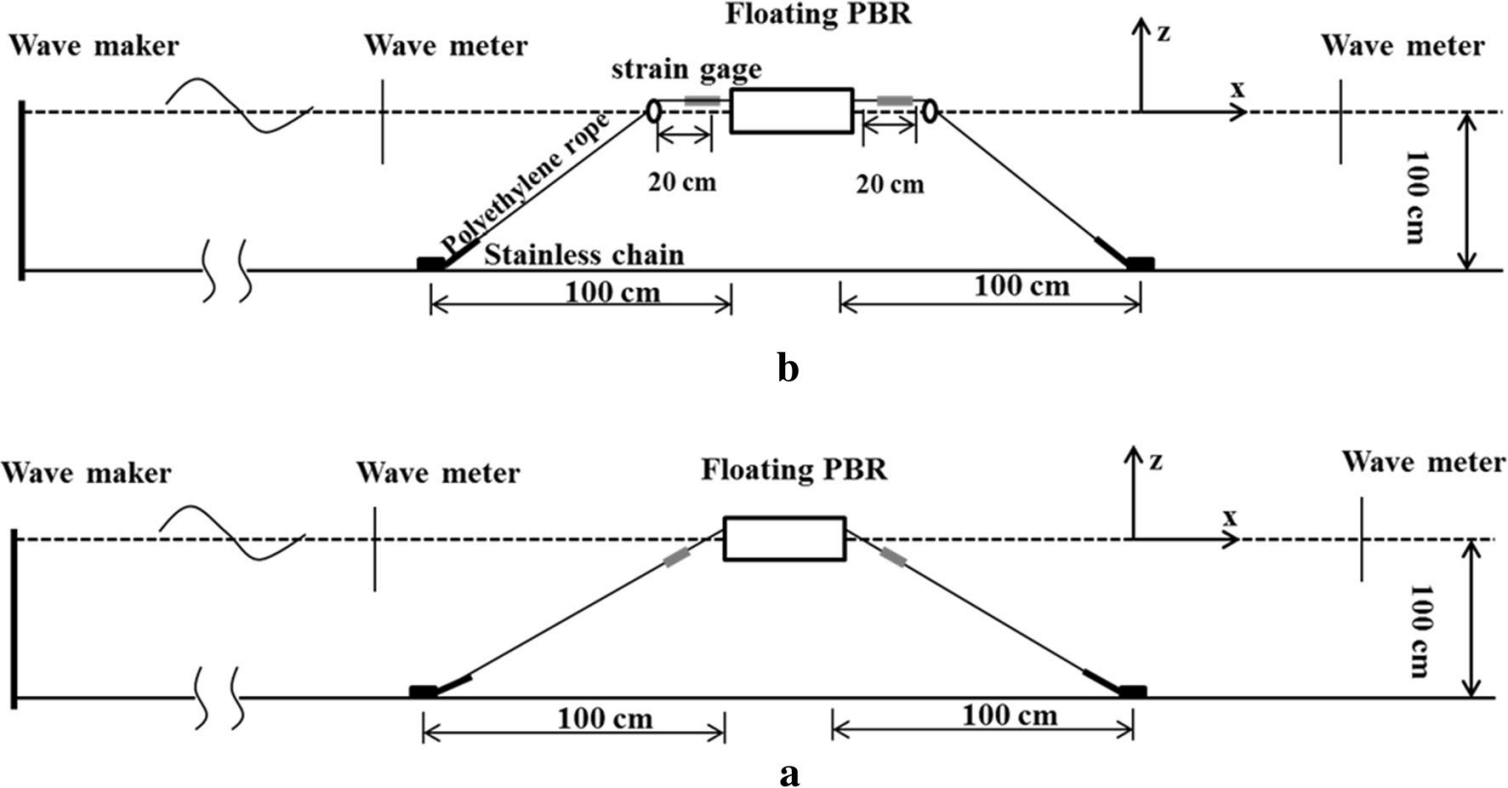
Diagrams of the experimental setups: **a** mooring system with floaters and **b** without floaters

Figure 3 includes sketches illustrating two types of anchoring methods: one that does not utilize floaters (mode 1) and another that utilizes floaters (mode 2) on the mooring lines. The lengths of the sides of the square and rectangular PBRs that are perpendicular to the waves are 1.12 m and 0.72 m, respectively. Each floating PBR was slack-moored in its equilibrium position using four mooring lines. Each mooring line consisted of one 121.4-cm polyethylene rope and one 20.0 -m stainless-steel chain. The line densities of the rope and the chain are 0.003 kg/m and 0.13 kg/m, respectively. To measure the forces acting on the mooring lines, four load cells were connected to the windward and the leeward mooring lines. Two diodes were fixed on the floating PBR for the purposes of movement analysis. A charge-coupled device (CCD) camera was used to record the movement trajectories of the diodes. The software DUT-FlexSim [36], which was developed in-house, was used to calculate the movement responses of the floating PBRs.

### Experimental wave conditions

Regular waves were studied at a prototype water depth of 10.0 m. For regular waves, the prototype wave heights (*H*_p_) were 0.2, 0.4, 0.6, 0.8 and 1.0 m; the prototype wave period (*T*_p_) ranged from 2.53 to 5.69 s. According to the experimental model scales and Eqs. 1 and 2, the experimental wave periods (*T*) were determined to range between 0.8 and 1.8 s, and the experimental wave heights (*H*) were determined to range between 2.0 and 10.0 cm. Additional information regarding the wave parameters is presented in Table 2.

**Table 2:** Experimental test conditions: wave height and period for the model and the prototype

*H*_p_ (m)	*T*_p_ (s)	*H*_m_ (cm)	*T*_m_ (s)
0.2	3.16	2	1.0
0.4	3.16	4	1.0
0.6	2.53, 3.16, 3.79, 4.43, 5.06, 5.69	6	0.8, 1.0, 1.2, 1.4, 1.6, 1.8
0.8	3.16	8	1.0
1.0	2.53, 3.16, 3.79, 4.43, 5.06, 5.69	10	0.8, 1.0, 1.2, 1.4, 1.6, 1.8

*H*_p_ and *T*_p_ is prototype wave height and wave period, respectively, while *H*_m_ and *T*_m_ is model wave height and wave period

In addition, the effect of the culture depth on the movement and mooring force was determined in the same wave conditions. In this study, three culture depths (0.5, 1.0 and 2.0 cm) were used, during which 5-, 10- and 20-L water was pumped into the PBR via the sampling port, respectively; the ratio of the culture mass to the PBR mass for each of the three conditions was 8.62, 17.2 and 34.5, respectively. According to the experimental model scales, the corresponding prototype culture depths for the floating PBRs were 5.0, 10.0 and 20.0 cm, respectively, which have been widely utilized during production in floating PBRs [16, 19].

### Measurements and data analysis method

In this study, three parameters were determined to characterize the movement responses of the floating PBR: movement, amplitude, and the roll angle. The movements were classified in terms of vertical and horizontal movement, including forward and backward movement, while the amplitude was determined from differences between the forward and backward movement.

To measure the motion of the PBRs, a CCD image-scanning technique was adopted that was based on that of Gui et al. [36], which is a reliable and efficient approach for the study of the motion responses of moving objects. The technique is an optical method and mainly consists of the following steps: (i) acquisition of a continuous image, (ii) identification and tracking of tracing points, (iii) coordinate calibration of the image, (iv) determination of the gray threshold, (v) scanning of the trajectories, and (vi) data analysis. To record the positional changes of the PBRs, two white light-emitting diode bulbs with a distance of 20 cm were installed on the side of PBR and a digital camera was used to continuously capture images at a rate of 0.05 s per image over a period of 10 s; a total of 180 images were collected for data analysis. The algorithm used for data analysis was presented in our previous study [36]. In addition, prior to capturing the images of the PBRs, the regular wave that was produced was verified to be stable using capacitance-type wave gauges that were arranged along the centerline of the wave flume; the absolute accuracy of these wave gauges is approximately ± 1 mm.

In addition, the force on the mooring line was also determined, where water-resistant load cells with a capacity of 10 N were connected to the mooring line at one site and to the PBR at another site, as shown in Fig. 3. The specified accuracy of the load cell is 0.001 N. Each measurement was made once and 180 continuous data points were collected during 10 s of data processing. The peak data were used as the present data.

## Results and discussion

### Effects of wave height and period on the hydrodynamic movement of floating PBRs

**Fig. 4:**
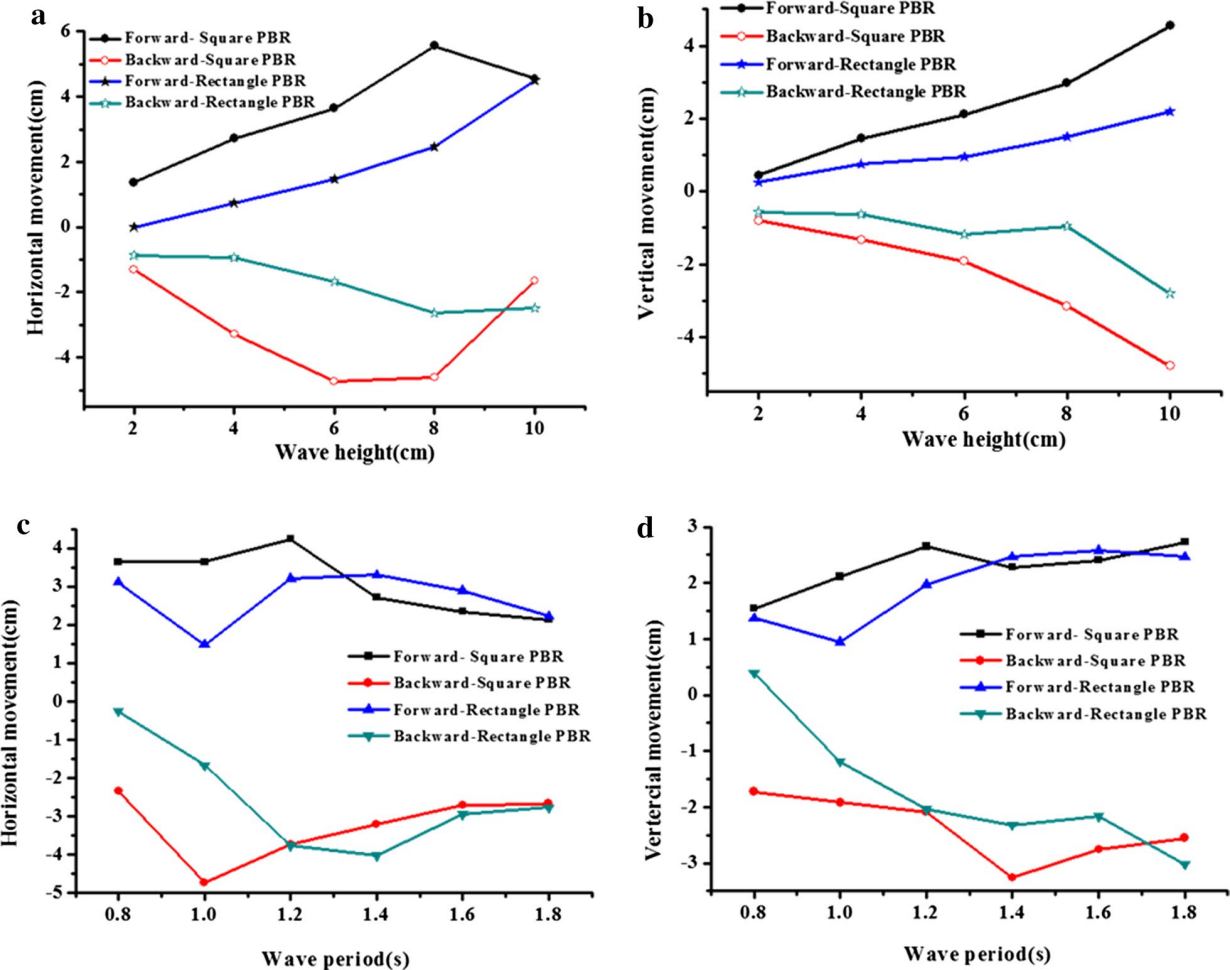
The movement of PBRs in response to different wave conditions: **a**, **c** horizontal movement; **b**, **d** vertical movement

**Fig. 5:**
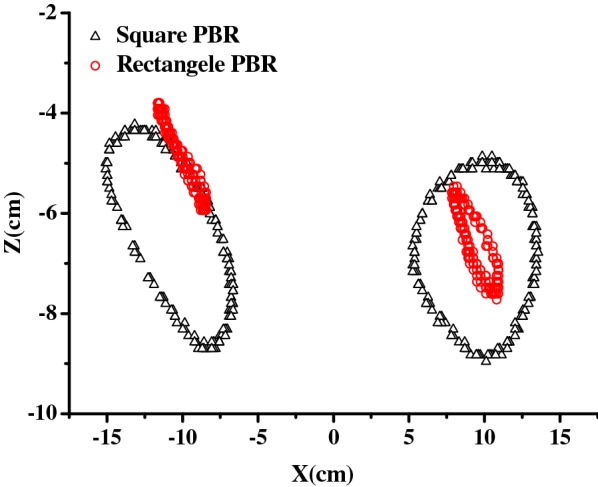
Trajectory of the two tracer points of the PBR models (*H* = 6 cm, *T* = 1 s)

Figure 4 shows the movement responses of the floating PBRs in waves. Based on the position of the PBR in still water as a reference, the maximum forward and backward movements are presented with respect to the wave height and wave period, respectively. Overall, both the horizontal and vertical movements of the PBRs increased with increasing wave height (Fig. 4a, b). The square model experienced more intensive movement than the rectangular model at the tested wave heights, during which the square PBR experienced horizontal movement ranging from − 4.73 to 5.56 cm and vertical movement ranging from − 4.80 to 4.55 cm; whereas, the rectangle PBR experienced horizontal movement ranging from − 2.65 to 4.5 cm and vertical movement ranging from − 2.80 to 2.19 cm. This finding was consistent with the results of the movement trajectories, which showed that the square model had a wide range of movement trajectories (Fig. 5). These results indicated that PBR geometry can significantly affect floating PBR hydrodynamics.

In studies of movement in breakwater, similar results were also observed. The most intensive movement was observed in breakwater with a *B*/*L* of 0.15 (*B* is the width of the breakwater and *L* is the wave length) [37]. This difference is likely caused by both the step size of wave and the aspect ratio of the floating PBR, which will be investigated in future studies. Thus, intensive movement of PBRs can be attained via optimization of the PBR aspect ratio (length and width). Additionally, the aspect ratio is of significance during PBR designing, as length and width determine the effective area of the PBR. Hence, a larger-scale PBR that is subject to intense movements can be achieved via further investigation of its design. For instance, a 100 m^2^ floating platform is being constructed based on the square PBR model and will be the largest closed PBR in the world. More importantly, a PBR at this scale will have great advantages in terms of reducing the production costs of microalgae biofuels for the following reasons: (1) the labor costs can be significantly reduced at this large scale; (2) the process of culture inoculation, biomass harvesting and oil extraction can be operated on this platform in the ocean, which will reduce the costs of PBR transportation between the ocean and the wharf; (3) to reduce the costs of nutrients and harvesting, a membrane filter can be installed to produce freshwater and nutrients that can also be used to harvest biomass, which has been established to be a cost-effective method of biomass harvesting [38] (a cell sedimentation pipette can be designed and connected to the bottom of the PBR can be designed, but this will require further study); and (4) wave power and solar power devices can be installed on the floating platform to provide power for processes required for culture pumping, mixing, biomass harvesting and oil extraction, and thus, the microalgal biofuel production will be completely driven by renewable energy, which will have a positive effect on the net energy ratio of the microalgae biofuel production.

**Fig. 6:**
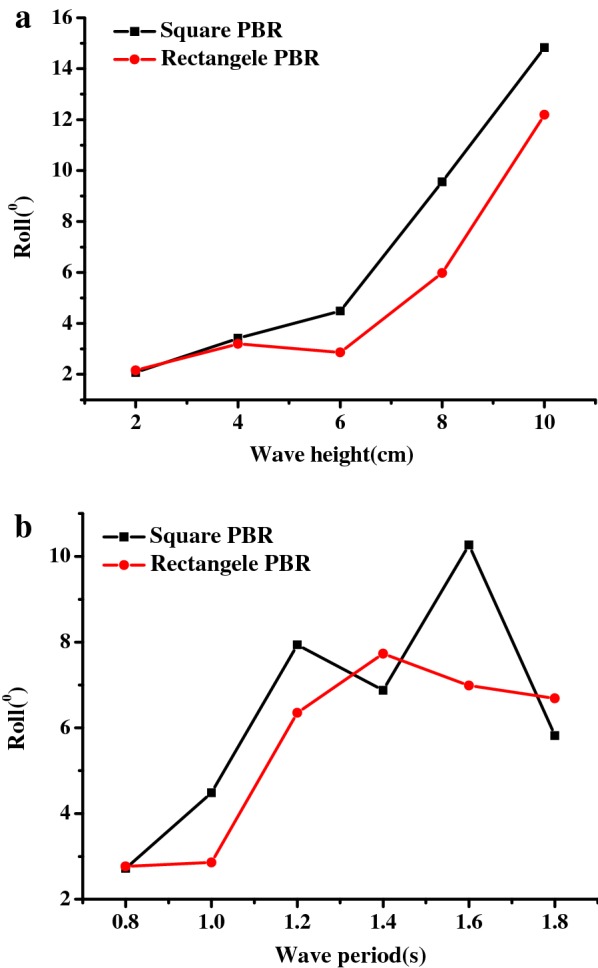
Inclination movement of the square and rectangular PBRs in response to different wave conditions: **a** wave height (*T* = 1.0 s); **b** wave period (*H* = 6 cm)

Compared with the wave height, the effects of the wave period on PBR movement were more complex. As shown in Fig. 4c, the horizontal movement of the PBR increased at first and then decreased as the wave period increased. The vertical movement of the PBR continued to increase as the wave period increased for short and medium period waves (*T* < 1.4 s), but there was no noticeable variation for long period waves (Fig. 4d). Additionally, the square model experienced more intensive movement than the rectangular model at the tested wave periods. The square PBR experienced horizontal movement ranging from − 4.73 to 4.23 cm and vertical movement ranging from − 3.26 to 2.72 cm, whereas the rectangular PBR experienced horizontal movement ranging from − 4.02 to 3.30 cm and vertical movement ranging from − 3.02 to 2.58 cm, respectively. The roll movement showed a similar trend for the tested wave periods, during which it increased to a maximum value of 7.9° for the square model and 7.7° for rectangular model, and then followed a downward trend (Fig. 6b). In contrast, the roll movement grew monotonically with increasing wave height (Fig. 6a), which is considered to be related to the wave steepness.

Unlike other floating cultivation systems [26], mixing in floating PBRs that do not have a mixing device is driven only by wave movements [16, 19]. The results of this study showed that a PBR with dimensions of 10 m × 10 m can exhibit horizontal movement ranging from − 47.3 to 55.6 cm and vertical movement ranging from − 48.0 to 45.5 cm at a wave height of 0.2–1.0 m, a wave period of 2.53–5.69 s, and a roll movement of 2.01°–14.83°. In response to movements in these ranges, intensive mixing can be predicted; for example, an orbital shaker or wave-generating shaker with a rocking angle of 2–10° has a mixing time ranging from of 18 to 71 s and a k_L_a_CO2_ of 9–66 h^−1^ [39]. It has been reported that a k_L_a_CO2_ of 2.95–17.7 h^−1^ has been reported in other floating PBRs [26], and the highest known value of k_L_a_CO2_ (3.93 h^−1^) was also reported for a BICCAPSO that was driven by artificial waves [16], which assumes that the ratio of k_L_a_CO2_ to k_L_a_O2_ is 0.983 at a temperature of 25 °C (according to Gaoxi et al.) [11]. In a major offshore area, the average wave height ranges from 0.2 to 1.0 m and the average wave period ranges from 2.1 to 4.5 s. It can be predicted that the 100 m^2^ floating PBR driven by natural wave can has the same level of k_L_a_CO2_ as the wave-generating shaker, which was higher than the two aforementioned floating PBRs, but this will be determined with experimental method in future.

Photosynthetic efficiency in a floating PBR depends mainly upon its fluid dynamics (or liquid sloshing), as this determines the light distribution, mixing performance, and nutrient mass transfer [21, 40]. This study investigated only the movements of floating PBRs. To date, liquid sloshing in an LNG-FPSO ship has been intensely studied using computational fluid dynamics (CFD) simulations [33, 34, 41], and CFD simulation has also been widely used for the modeling of PBRs [42, 43]. However, for the CFD simulation study of floating PBRs, the movement parameters, including the amplitudes of the horizontal and vertical movements and the roll movement (Eqs. 1 and 2), must first be experimentally determined; this is the reason that we conducted the present study. For the next step, the excitation frequency of the culture in floating PBRs will also be investigated according to a method that has been widely used in the studies of ships with liquid tankers, which are subject to the same phenomenon of liquid sloshing as floating PBRs on the ocean [34, 44]. Thus, a better understanding of the inner fluid dynamics of floating PBRs, as characterized by k_L_a, the mixing time, and the light–dark recycle, can be achieved using CFD simulation based on data from the present results. The inner structure of a PBR can be redesigned and optimized to induce more efficient culture mixing using CFD [41].

1 $$S_{x} (t) = A \, \cos \theta \, \sin \, \omega t,$$

2 $$S_{y} (t) = A \, \sin \theta \, \sin \, \omega t,$$

where *A* is the movement amplitude of the horizontal and vertical reactions, *θ* is the amplitude of the roll movement, *ω* is the excitation frequency of culture in PBR, and *t* is time.

### Effects of wave height and period on PBR mooring forces

**Fig. 7:**
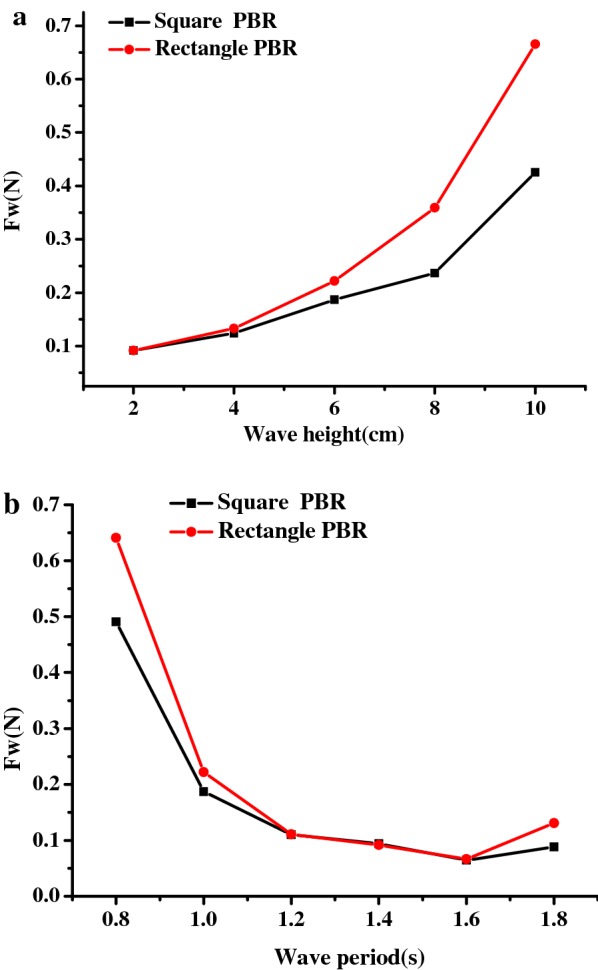
Forces acting on windward mooring lines of the square and rectangular PBR models in response to different wave conditions: **a** wave height (*T* = 1.0 s); **b** wave period (*H* = 6 cm)

To ensure the safe operation of the PBRs, the forces acting on the mooring lines are important parameters to be considered for the design of mooring system. Because the windward mooring lines always withstand more force than the leeward lines in waves, only the forces acting on the windward mooring lines were analyzed for the square model and the rectangular model. As shown in Fig. 7a, the forces acting on the square model and the rectangular model both increased monotonically with increasing wave height and a constant wave period, during which the highest mooring force (0.43 N) was measured for the square PBR, whereas that of the rectangular PBR was 0.67 N. Based on to these results, the calculated highest mooring forces for the 100 m^2^ square and rectangular PBRs were 430 and 670 N, respectively. It has been reported that the highest mooring line forces (0.94 N) are found in floating cages [45], which have been widely used for fish culture and can be safely used even in undersea conditions. Compared to this, the mooring forces for the floating PBRs were low. Thus, an offshore microalgae plant could be safely used as a floating cage.

Compared with effect of the wave height, the forces acting on the PBR decreased dramatically for short wave periods and were maintained at a nearly constant level for medium and long wave periods, with a slight increase for the largest wave period (*T* = 1.8 s; Fig. 7b). Overall, the square model experiences greater wave loads than the rectangle model during most of the studied scenarios, especially for waves with large wave steepness, which corresponds to a high wave height and a short wave period.

### Effects of culture depth

**Fig. 8:**
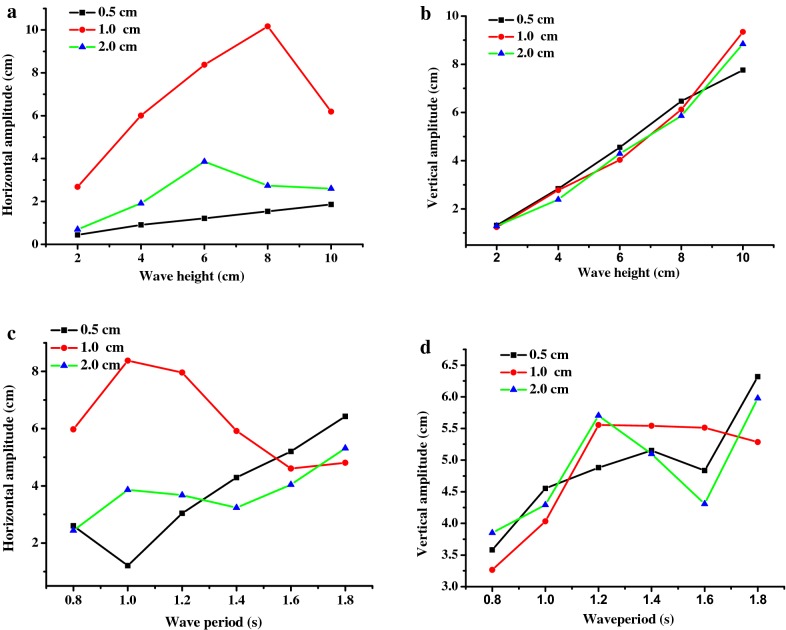
Effects of culture depth on the amplitudes of the square PBR model in response to different wave conditions: **a** and **b** wave height (*T* = 1.0 s); **c** and **d** wave period (*H* = 6 cm)

Culture depth is a significant parameter for microalgae cultivation [46] and the movement of floating PBRs. In this study, three depths (0.5, 1.0, and 2.0 cm) were selected, which correspond to 5.0, 10, and 20 cm during practical cultivation. As shown in Fig. 8a, c, there were significant differences in the horizontal amplitude of the square PBR at different culture depths, and the most intense amplitude was found at a depth of 1.0 cm for short and medium period waves, during which the highest horizontal and vertical amplitudes were 10.17 cm and 9.35 cm, respectively. Differences were also observed for the vertical amplitude, though only slight differences were observed at the tested wave heights and a constant wave period of 1.0 s (Fig. 8b). Similar results were also observed for the rectangular model (data not shown), indicating that water depth can significantly affect the amplitude of floating PBRs. These differences are likely caused by the resonance frequency, where the produced frequency of the PBR with a 1-cm culture depth was the same as its resonance frequency. This topic will be studied experimentally in the future. As mixing in the floating PBRs was induced by only wave movement, there must be great differences in the fluid dynamics of PBRs at different culture depths, including mixing characteristics and flashing light effects. These difference were observed in our studies [47]. To better understand these differences, CFD will be used to simulate the hydrodynamics and mixing behavior of floating PBRs at different culture depths in the future.

**Fig. 9:**
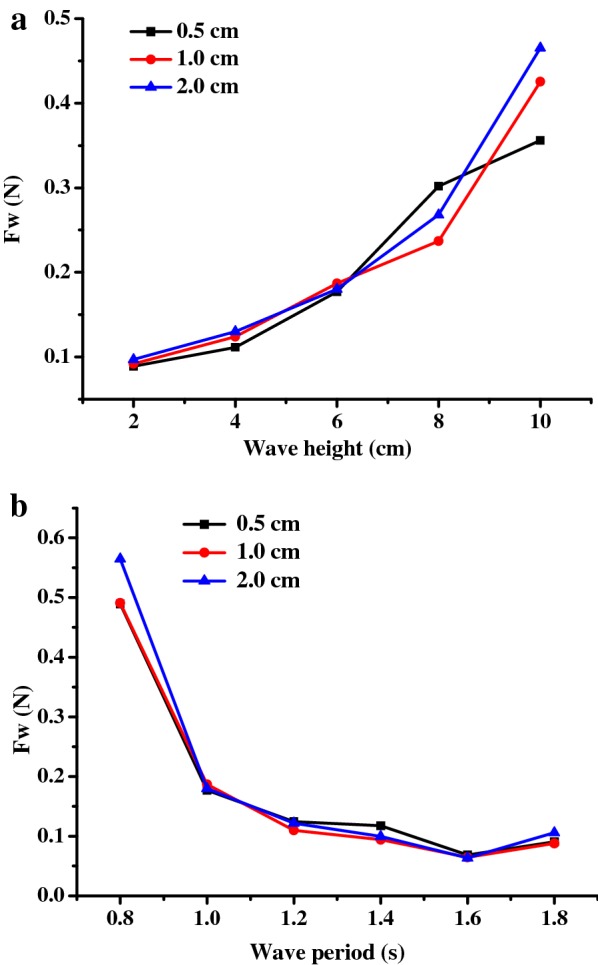
Effects of culture depth on mooring forces in the square PBR model in response to different wave conditions: **a** wave height (*T* = 1.0 s); **b** wave period (*H* = 6 cm)

Changes in the mooring forces in response to different culture depths were also measured. As shown in Fig. 9a, b, the results showed that there were no statistically significant differences between the culture depths used for the square model within the studied range of waves and the forces acting on the PBR, which increased monotonically with increasing wave height and decreased with increasing wave period at the studied water depths. Similar results were also observed for the rectangle model. The results indicated that the depth of the water column has no noticeable effect on the forces acting on the PBRs, but the explanation for this was not clear.

### Effects of the mooring system on PBR movement and mooring forces

Unlike conventional cultivation systems constructed on land, floating PBRs are significantly affected by the mooring systems that are used to ensure their safe operation. In this study, two different types of mooring system (with/without floater) were designed (Fig. 3). According to the results of the laboratory experiments, the movement responses of the PBR show no statistically significant differences when either of two different mooring types were used (data not shown). This indicates that the added floaters in the mooring system will have no significant effect on the movement of the PBR, as its movement is affected primarily by the water depth of the PBR and its structure.

**Fig. 10:**
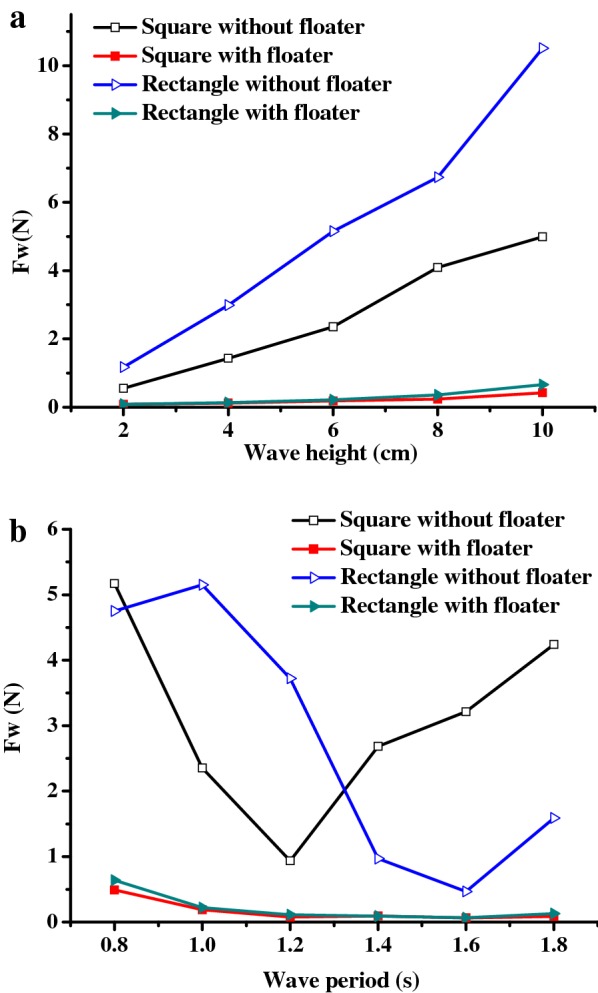
Forces acting on the windward mooring lines of the square and rectangular PBRs with different anchoring modes: **a** wave height (*T* = 1 s); **b** wave period (*H* = 0.06 cm)

However, the forces acting on the mooring lines of both the square model and the rectangular model of the PBRs were dramatically reduced when floaters were attached to the mooring lines. As shown in Fig. 10, the highest mooring forces for the square and the rectangular PBRs without floaters were 5.0 and 10.5 N, respectively; whereas that of the PBRs with floaters were only 0.43 and 0.67 N, respectively. It is thought that the floaters can result in a certain buoyancy and movement due to changing wave elevation, which can reduce the peak value of the wave load that acts on the mooring line due to the cushioning effects. Thus, a mooring system with floaters is recommended for use with a floating PBR for practical reasons. In addition, these results also provide guidance for PBR manufacturing materials. For the prototype 100 m^2^ PBR, the maximum tolerance of the materials and belts that are connected to the mooring system should be greater than 0.67 kN, based on the results of the square model. Apart from the mooring system, the arrangement of floating PBRs can also have significant effect on their movement and the acting mooring forces [48], which will be studied in the future.

This study resulted in a report on the hydrodynamic movement of floating PBRs in response to waves. Results have shown that movement was affected by the wave conditions (wave height and period), culture depth, and PBR configuration. Comparatively, the wave conditions had a predominant effect, especially the wave height, as waves provided the sole source of energy for mixing. The intense movement of floating PBRs can only be achieved via optimization of the culture depth and PBR construction in the presence of sufficient wave energy. However, waves are uncontrollable due to wave intermittence, which limits the application of floating PBRs during times and in places subject to low wave height, as serious negative effects on algae growth may occur due to the accumulation of high levels of dissolved oxygen [16]. When algae growth in floating PBRs was tested in the ocean, the highest biomass productivity (18.9 g m^−2^ day^−1^) was achieved [19], although the wave height was almost zero during some sunny days. This result indicated that low wave height may have no effect on algae growth, but this requires further investigation.

Certainly, oceans with high wave heights are ideal, as this will provide sufficient energy to shake the floating PBRs. In this study, there was no overtopping at all tested wave heights (from 2 to 10 cm); thus, the PBRs should be safe if they are deployed in an ocean field with a wave height of less than 1.0 m. On the other hand, the effects of destructive forces should be considered, as well as the security issues that other ocean engineering equipment often faces. To avoid this, the selection of a proper mooring system is significant, as a mooring system containing floaters can protect the floating PBR from avulsion due to high mooring forces. In addition, an anti-wave floating PBR could be designed to utilize wave energy more efficiently in the deep ocean. This floating PBR could be developed using designs of floating cages [45]; this will be investigated further in the future.

## Conclusions

The hydrodynamic movements of floating PBRs in response to wave conditions were reported. The results showed that the movement of floating PBRs increased with increasing wave height and decreased with increasing wave period. Additionally, the configuration and culture depths used for PBRs have significant effects on their hydrodynamic movement, which demonstrated that a square PBR model with a 1.0-cm depth experienced more intense movement than a rectangular PBR model, but also experienced little mooring force. In addition, the mooring system has no effect on the hydrodynamic performance of a floating PBR, but the use of a mooring system with floaters can significantly decrease mooring line forces, which indicates that a mooring system can effectively protect floating PBRs from destruction by waves.

## Abbreviations

PBR: photobioreactor
CFD: computational fluid dynamics
BICCAPSO: bicarbonate-based carbon capture and algal production system on ocean
PVC: polyvinyl chloride
CCD: charge-coupled device
LNG-FPSO: liquefied natural gas-floating production, storage and offloading system

## References

[1] Tredici MR (2010). Photobiology of microalgae mass cultures: understanding the tools for the next green revolution. Biofuels.

[2] Zhu C (2018). A recycling culture of *Neochloris oleoabundans* in a bicarbonate-based integrated carbon capture and algae production system with harvesting by auto-flocculation. Biotechnol Biofuels.

[3] Darvehei P, Bahri PA, Moheimani NR (2018). Model development for the growth of microalgae: a review. Renew Sustain Energy Rev.

[4] Xu K (2018). Toward the lowest energy consumption and emission in biofuel production: combination of ideal reactors and robust hosts. Curr Opin Biotechnol.

[5] Sims RE (2010). An overview of second generation biofuel technologies. Bioresour Technol.

[6] Barsanti L, Gualtieri P (2018). Is exploitation of microalgae economically and energetically sustainable?. Algal Res.

[7] Kenny P, Flynn KJ (2017). Physiology limits commercially viable photoautotrophic production of microalgal biofuels. J Appl Phycol.

[8] Hoffman J (2017). Techno-economic assessment of open microalgae production systems. Algal Res.

[9] Norsker N-H (2011). Microalgal production—a close look at the economics. Biotechnol Adv.

[10] Ruiz J (2016). Towards industrial products from microalgae. Energy Environ Sci.

[11] Xi G, Bo K, Vigil RD (2015). Characteristic time scales of mixing, mass transfer and biomass growth in a Taylor vortex algal photobioreactor. Bioresour Technol.

[12] Chang H (2018). High-efficiency nutrients reclamation from landfill leachate by microalgae *Chlorella vulgaris* in membrane photobioreactor for bio-lipid production. Bioresour Technol.

[13] Chang H (2019). Microalgal lipids production and nutrients recovery from landfill leachate using membrane photobioreactor. Bioresour Technol.

[14] Xi G, Bo K, Vigil RD (2018). Simulation of algal photobioreactors: recent developments and challenges. Biotechnol Lett.

[15] Qin C, Wu J (2019). Influence of successive and independent arrangement of Kenics mixer units on light/dark cycle and energy consumption in a tubular microalgae photobioreactor. Algal Res.

[16] Zhu C (2018). Bicarbonate-based carbon capture and algal production system on ocean with floating inflatable-membrane photobioreactor. J Appl Phycol.

[17] Willson B, et al. Diffuse light extended surface area water-supported photobioreactor. 2008.

[18] Kim ZH (2015). Algal biomass and biodiesel production by utilizing the nutrients dissolved in seawater using semi-permeable membrane photobioreactors. J Appl Phycol.

[19] Zhu C (2018). Large-scale cultivation of *Spirulina* in a floating horizontal photobioreactor without aeration or an agitation device. Appl Micro Biotechnol..

[20] Zhu C (2018). Seawater desalination concentrate for cultivation of *Dunaliella salina* with floating photobioreactor to produce beta-carotene. Algal Res.

[21] Abu-Ghosh S (2016). Flashing light in microalgae biotechnology. Bioresour Technol.

[22] Gao X, Kong B, Vigil RD (2018). Multiphysics simulation of algal growth in an airlift photobioreactor: effects of fluid mixing and shear stress. Bioresour Technol.

[23] Jorquera O (2010). Comparative energy life-cycle analyses of microalgal biomass production in open ponds and photobioreactors. Bioresour Technol.

[24] Kumar K (2015). Recent trends in the mass cultivation of algae in raceway ponds. Renew Sustain Energy Rev..

[25] Wijffels RH, Barbosa MJ (2010). An outlook on microalgal biofuels. Science.

[26] Kim ZH (2016). Development of a floating photobioreactor with internal partitions for efficient utilization of ocean wave into improved mass transfer and algal culture mixing. Bioprocess Biosyst Eng.

[27] Pirasaci T (2017). Hydrodynamic design of an enclosed Horizontal BioReactor (HBR) for algae cultivation. Algal Res.

[28] Wiley P (2013). Microalgae cultivation using offshore membrane enclosures for growing algae (OMEGA). J Sustain Bioenergy Syst.

[29] Klein-Marcuschamer D (2013). A matter of detail: assessing the true potential of microalgal biofuels. Biotechnol Bioeng.

[30] Novoveska L (2016). Optimizing microalgae cultivation and wastewater treatment in large-scale offshore photobioreactors. Algal Res.

[31] Dogaris I (2015). A novel horizontal photobioreactor for high-density cultivation of microalgae. Bioresour Technol.

[32] Dong GH (2008). Experiments on wave transmission coefficients of floating breakwaters. Ocean Eng.

[33] Shi Y (2017). Experimental studies on performances of flexible floating oil booms in coupled wave-current flow. Appl Ocean Res.

[34] Jiang SC (2015). Numerical simulation of coupling effect between ship motion and liquid sloshing under wave action. Ocean Eng.

[35] Ji CY (2015). Experimental study of a new type of floating breakwater. Ocean Eng.

[36] Gui F (2006). Application of CCD image scanning to sea-cage motion response analysis. Aquacult Eng.

[37] Mani JS (1991). Design of Y-frame floating breakwater. J Waterw Port Coast.

[38] Fasaei F (2018). Techno-economic evaluation of microalgae harvesting and dewatering systems. Algal Res.

[39] Jones SMJ, Louw TM, Harrison STL (2017). Energy consumption due to mixing and mass transfer in a wave photobioreactor. Algal Res.

[40] Pires JCM, Alvim-Ferraz MCM, Martins FG (2017). Photobioreactor design for microalgae production through computational fluid dynamics: a review. Renew Sustain Energy Rev.

[41] Cheng J (2017). Alternatively permutated conic baffles generate vortex flow field to improve microalgal productivity in a raceway pond. Bioresour Technol.

[42] Gao X, Kong B, Vigil RD (2017). Comprehensive computational model for combining fluid hydrodynamics, light transport and biomass growth in a Taylor vortex algal photobioreactor: Eulerian approach. Algal Res.

[43] Gao X, Kong B, Vigil RD (2017). Comprehensive computational model for combining fluid hydrodynamics, light transport and biomass growth in a Taylor vortex algal photobioreactor: Lagrangian approach. Bioresour Technol.

[44] Yan S, Liu ZY (2017). Coupling effects of barge motion and sloshing. Ocean Eng.

[45] Xu T, et al. Hydrodynamic characteristics of floating pipes in random waves. In: 32nd ASME international conference on ocean, vol. 5. 2013.

[46] Bechet Q, Shilton A, Guieysse B (2013). Modeling the effects of light and temperature on algae growth: state of the art and critical assessment for productivity prediction during outdoor cultivation. Biotechnol Adv.

[47] Zhu H (2017). Plastic bag as horizontal photobioreactor on rocking platform driven by water power for culture of alkalihalophilic cyanobacterium. Bioresour Bioprocess.

[48] Xu TJ (2013). Analysis of hydrodynamic behavior of a submersible net cage and mooring system in waves and current. Appl Ocean Res.

